# Decreased Interleukin-4 Release from the Neurons of the Locus Coeruleus in Response to Immobilization Stress

**DOI:** 10.1155/2016/3501905

**Published:** 2016-01-19

**Authors:** Hyun-ju Lee, Hyun-Jung Park, Angela Starkweather, Kyungeh An, Insop Shim

**Affiliations:** ^1^Acupuncture and Meridian Science Research Center, College of Oriental Medicine, Kyung Hee University, 26 Kyunghee-daero, Dongdaemun-gu, Seoul 130-701, Republic of Korea; ^2^Department of Adult Health and Nursing Systems, School of Nursing, Virginia Commonwealth University, Richmond, VA 23298-0567, USA

## Abstract

It has been demonstrated that immobilization (IMO) stress affects neuroimmune systems followed by alterations of physiology and behavior. Interleukin-4 (IL-4), an anti-inflammatory cytokine, is known to regulate inflammation caused by immune challenge but the effect of IMO on modulation of IL-4 expression in the brain has not been assessed yet. Here, it was demonstrated that IL-4 was produced by noradrenergic neurons in the locus coeruleus (LC) of the brain and release of IL-4 was reduced in response to IMO. It was observed that IMO groups were more anxious than nontreated groups. Acute IMO (2 h/day, once) stimulated secretion of plasma corticosterone and tyrosine hydroxylase (TH) in the LC whereas these increments were diminished in exposure to chronic stress (2 h/day, 21 consecutive days). Glucocorticoid receptor (GR), TH, and IL-4-expressing cells were localized in identical neurons of the LC, indicating that hypothalamic-pituitary-adrenal- (HPA-) axis and sympathetic-adrenal-medullary- (SAM-) axis might be involved in IL-4 secretion in the stress response. Accordingly, it was concluded that stress-induced decline of IL-4 concentration from LC neurons may be related to anxiety-like behavior and an inverse relationship exists between IL-4 secretion and HPA/SAM-axes activation.

## 1. Introduction

Stress induces neuroinflammation accompanied by altered production of neuropeptides and inflammatory cytokines in the central nervous system (CNS). Such modulation in the CNS affects endocrine and immune systems followed by behavioral changes [1–3]. Immobilization (IMO) is a severe stressor that triggers both physiological and behavioral responses.

An inflammatory process is developed by imbalanced release of pro- and anti-inflammatory cytokines in response to IMO. Interleukin-4 (IL-4) is an anti-inflammatory cytokine and has an ability to inhibit synthesis of IL-1*β*, a proinflammatory cytokine, and upregulate the production of IL-1 receptor antagonist [4, 5]. The effect of stress on secretion of IL-4 in the periphery is highly controversial. It has been shown that serum IL-4 level was decreased [6], and not changed [7], or the number of airway IL-4 positive cells was increased [8] in various animal models of stress. Several human studies reported that social stress test and public speaking had no association with blood IL-4 concentration [2, 9, 10]. Stress-induced modification of IL-4 release in the brain, however, has not been assessed fully. Th2 helper T lymphocytes are responsible for production of IL-4 in the periphery [11] and microglia are reported as a IL-4 secreting cell in the cortex of the brain [12], whereas IL-4 producing cells in the LC of the brain have not been identified yet.

Activation of the hypothalamic-pituitary-adrenal- (HPA-) and sympathetic-adrenal-medullary- (SAM-) axes is also a typical response induced by IMO. The final consequence of HPA-axis activation is increased corticosterone (CORT) release in the plasma [13–16]. The locus coeruleus (LC) is a stress-response brainstem and a major part of the SAM-axis for its role in regulating norepinephrine (NE) release. Secretion of NE and expression of tyrosine hydroxylase (TH), an enzyme involved in the synthesis of NE, are stimulated as a result of SAM-axis activation [17, 18]. SAM-axis modulation by the HPA-axis occurs via projections of the LC to the stress-related brain regions interconnected with the hypothalamus [19]. Anxiety-like behavior in the elevated plus maze (EPM) test was also reported in an IMO-administered animal group [20, 21]. Several researches have shown that glucocorticoid influences IL-4 production and IL-4 signaling* in vitro* [22, 23]. It was also shown that injection of IL-4 altered free radical processes evoked by stress [24]. Stress-induced involvement of IL-4 in HPA- and SAM-axes activation* in vivo* is not fully delineated yet, whereas the release of proinflammatory cytokines has been well established.

The purpose of this study was to assess the influence of acute and chronic IMO on IL-4 concentrations and to identify the characteristics of IL-4 producing cells in the LC region of the brain. We also focused on the correlation of IL-4 secretion, anxiety-like behavior, and HPA-/SAM-axes activation in the stress response.

## 2. Materials and Methods

### 2.1. Animals

All the experimental procedures performed on the animals were conducted with the approval of the Ethics Committee of the Kyung Hee University (KHUAP(SE)-13-041) and in accordance with the US National Institutes of Health (*Guide for the Care and Use of Laboratory Animals*, 8th edition, revised 2011). Male Sprague-Dawley rats (Orient Animal Corp., Gyeonggi-do, Korea) aged 7 weeks (310–360 g) were housed under a 12-h light schedule with controlled temperature at 22°C and humidity. Animals had access to water and food* ad libitum* and were acclimated for 7 days prior to experiments. The experiments were performed according to the animal care guidelines of the NIH and Kyung Hee University Institutional Animal Care.

### 2.2. Immobilization Stress Procedure

The animals were randomly assigned to control, acute IMO, or chronic IMO groups. The acute IMO group was restrained for 2 hours once in a cone-shaped PVC which restricts forward, backward, and lateral movements. Chronic IMO was administered daily for 21 consecutive days.

### 2.3. Elevated Plus Maze (EPM) Test

After exposure to stress, the animals were immediately tested in the EPM. The EPM test was adapted from Walf and Frye [25] except that the animals were placed in the center of the maze facing one of the closed arms. The time spent on the open arms and the closed arms of the maze were video-taped and recorded for 5 min by S-MART program (Pan-Lab, Barcelona, Spain).

### 2.4. Enzyme Linked Immunosorbent Assay (ELISA)

#### 2.4.1. Interleukin-4 (IL-4)

After all stress procedures were done, the animals were deeply anesthetized with sodium pentobarbital (80 mg/kg, administered i.p.) and the brains were immediately removed and sectioned in a coronal manner by using rodent brain matrix (ASI instruments Inc., MI, USA). The LC region of the brain was punched out on a cold plate and stored at −70°C until the assay. The obtained tissue was thawed and homogenized in ice cold cell lysis buffer (Cell Signaling Technology Inc., Danvers, MA, USA) and centrifuged (10,000 ×g at 4°C for 30 min). Protein concentrations in homogenates were equalized (1 *μ*g/*μ*L) by Bradford assay. IL-4 concentration in duplicate 100 *μ*L aliquots was assessed by ELISA kit according to the manufacturer's instructions (Promikine, Heidelberg, Germany).

#### 2.4.2. Corticosterone (CORT)

After exposure to the stress, the animals were anesthetized with sodium pentobarbital (80 mg/kg, administered i.p.) and cardiac blood was collected. The obtained sample was centrifuged (10,000 ×g at 4°C for 30 min) and plasma was collected and then stored at −70°C until the assay. Level of CORT in the plasma was analyzed by ELISA kits according to the manufacturer's instructions (Assay Designs, Ann Arbor, MI, USA).

#### 2.4.3. Immunofluorescent Staining

Double immunofluorescence staining of IL-4 with TH, released from noradrenergic neuron in the LC [26], glial fibrillary acidic protein (GFAP), an astrocyte specific marker, and ionized calcium-binding adaptor protein-1 (Iba-1), a marker for microglia, was performed to identify IL-4 secreting cell in the LC.

To determine the role of the HPA- and SAM-axes in IL-4 secretion via the LC during the stress response, glucocorticoid receptor (GR), TH, and IL-4-expressing cells were also double stained. The rat brains were removed after transcardial perfusion with 4% solution of formaldehyde (Sigma-Aldrich St. Louis, MO, USA), then postfixed in the same fixative for 24 hours, and placed in PBS containing 20% of sucrose for 72 hr. Serial coronal sections were cut into 30 *μ*m thickness by using a cryostat microtome (CM 1850UV, Leica Microsystems Inc., Wetzlar, Germany) and the sections were processed as free-floating. The sections of the LC (bregma −9.57 mm to −9.99 mm) were blocked with 10% v/v normal horse serum (Vector Laboratories, Inc., Burlingame, CA, USA) for 1 hr at room temperature with constant agitation at 100 rpm. The sections were then rinsed in PBS followed by incubation in IL-4 mouse monoclonal IgG (diluted 1 : 25, Santa Cruz Biotechnology, Inc., Dallas, Texas, USA), TH rabbit monoclonal IgG (diluted 1 : 1000, Millipore, San Francisco, CA, USA), Iba-1 rabbit polyclonal IgG (diluted 1 : 200, Wako, Japan), GFAP rabbit polyclonal IgG (diluted 1 : 2000, Abcam, Cambridge, UK), or GR rabbit polyclonal IgG (diluted 1 : 50, Santa Cruz Biotechnology, Inc., Dallas, Texas, USA) for 48 hours at 4°C with constant agitation. Then, the sections were rinsed in PBS and subsequently incubated with horse anti-mouse conjugated to fluorescein isothiocyanate (FITC) (diluted 1 : 200, Vector Laboratories, Inc., Burlingame, CA, USA) or fluorescent Alexa Fluor 546 dye-labeled anti-rabbit IgG at room temperature for 2 hours with constant agitation. The sections were again rinsed in PBS, mounted onto slides, and cover-slipped with Vectashield mounting medium (Vector Laboratories, Inc., Burlingame, CA, USA). Samples were viewed by confocal microscope (LSM 510 Meta, Carl Zeiss Inc., Oberkochen, Germany). The density of immunopositive neurons in the LC region was quantified according to Paxinos et al. [27] using the Scion image program (Scion Corp., MD, USA).

### 2.5. Statistics Analysis

The data were expressed as the mean ± standard error of the mean (SEM). Comparisons among different groups were analyzed by one-way analysis of variance (ANOVA) followed by Scheffe's test as a* post hoc* test. All the statistical analyses were performed using SPSS (version 18.0., SPSS, Chicago, IL, USA). *P* values below 0.05 were regarded as statistically significant.

## 3. Results

### 3.1. Elevated Plus Maze

**Figure 1:**
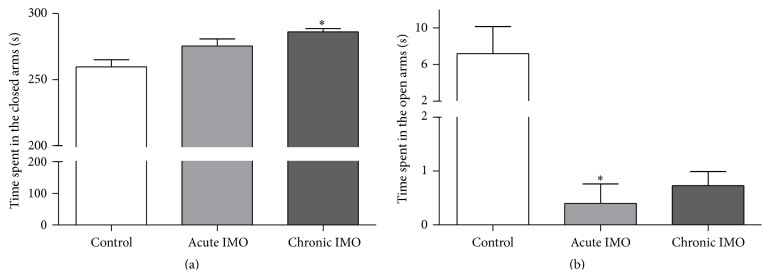
Mean (± SEM) time spent in the closed arms and the open arms during EPM test for the acutely and chronically stressed and control rats. (a) Chronic IMO induced a significant increase in time spent on the closed arms. (b) Acute IMO led animals to stay for significantly less amount of time in the open arms. (*n* = 6 per group) ^*∗*^ *P* < 0.05 versus control; ANOVA.

The EPM test was performed to compare the anxiety level between stressed and control rats. The time spent on the closed arms (*F*_2,13_ = 6.9, *P* < 0.05) and on the open arms (*F*_2,17_ = 4.9, *P* < 0.05) was recorded during the 5 minutes of the test (Figure 1). The chronic IMO group showed significantly increased time spent on the closed arms (*P* < 0.05; Figure 1(a)) and the acute IMO group had a less amount of time on the open arms (*P* < 0.05; Figure 1(b)) than the control group.

### 3.2. Enzyme Linked Immunosorbent Assay (ELISA)

#### 3.2.1. Interleukin-4 (IL-4)

**Figure 2:**
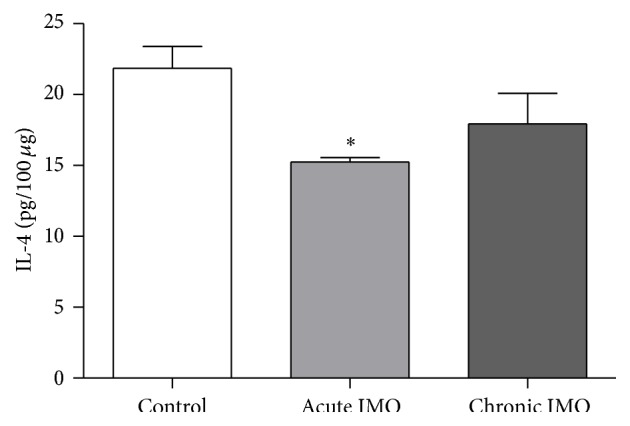
Mean (± SEM) IL-4 protein level in the LC of the acutely and chronically stressed and control rats. Expression of IL-4 was significantly decreased in the acutely stressed group. (*n* = 6 per group) ^*∗*^ *P* < 0.05 versus control; ANOVA.

Concentration of IL-4 protein was assessed in the 100 *μ*g aliquot of the LC in the IMO-subjected and nontreated rats. IMO had an effect on the decrease of IL-4 secretion in the rat brain stem (*F*_2,12_ = 6.9, *P* < 0.05; Figure 2). The rats under acute IMO showed significantly greater reduction in the release of IL-4 protein compared to the control group (*P* < 0.05).

#### 3.2.2. Corticosterone (CORT)

**Figure 3:**
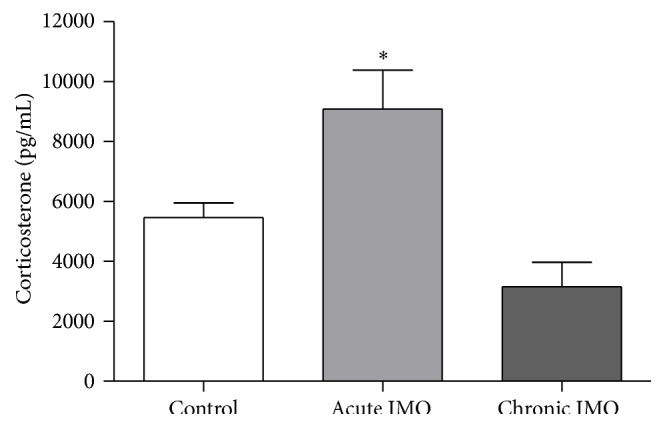
Mean (± SEM) CORT level in the plasma of the control, acute, and chronic IMO-submitted rats. Acute IMO significantly increased plasma CORT whereas chronic IMO induced reduction of CORT release. (*n* = 8 per group) ^*∗*^ *P* < 0.05 versus control; ANOVA.

Plasma CORT level was analyzed in the control, acutely and chronically stressed groups (*F*_2,24_ = 6.6, *P* < 0.05). The rats subjected to acute IMO revealed a significantly higher increment of CORT compared to controls (*P* < 0.05; Figure 3). In contrast, reduced release of CORT was showed in the chronic IMO group compared with the control group.

#### 3.2.3. Identification of IL-4-Producing Cells in the LC

**Figure 4:**
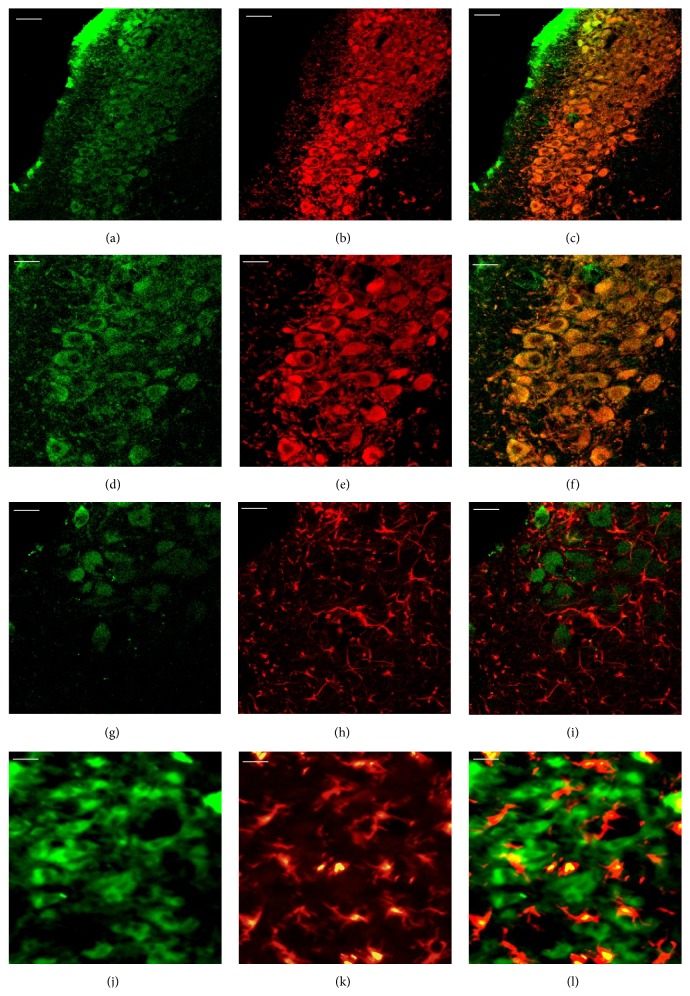
Characterization of IL-4-producing cells in the LC. (a–c) Representative fluorescent images showed that IL-4-secreting cells (a) were colocalized (c) with TH-releasing cells (b). Photomicrographs were taken at ×200 magnification.* Scale bars* = 50 *μ*m. (d–f) High power (taken at ×400 magnification) photographs of (a), (b), and (c), respectively, are shown.* Scale bars* = 20 *μ*m. IL-4-releasing cells (g and j) were not merged (i and l) with GFAP-positive cells (h) or Iba-1-immunoreactive cells (k). Photomicrographs were taken at ×400 magnification.* Scale bars* = 20 *μ*m.

Immunohistochemical analysis was used to characterize IL-4 releasing cells in the brain stem. Double staining revealed that IL-4-immunoreactive cells (Figures 4(a) and 4(d)) and TH-positive cells (Figures 4(b) and 4(e)) were colocalized in the LC (Figures 4(c) and 4(f)). IL-4 producing cells were not merged with immune-labeling of GFAP (an astrocyte specific marker) or Iba-1 (a marker for microglia) (Figures 4(i) and 4(l), resp.).

#### 3.2.4. Expression of GR

**Figure 5:**
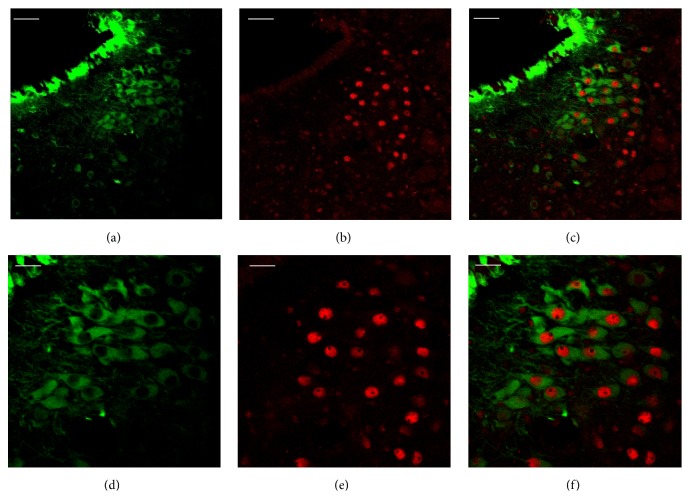
Localization of IL-4 and GR in the LC. (a–c) Representative fluorescent images showed that IL-4 (a) and GR (b) were expressed in the neurons but not in the identical part (c). Photomicrographs were taken at ×200 magnification.* Scale bars* = 50 *μ*m. (d–f) High power (taken at ×400 magnification) photographs of (a), (b), and (c), respectively, are shown.* Scale bars* = 20 *μ*m.

Immunofluorescent analysis was used to further explore the localization of GR and IL-4 in the LC. Double staining showed that IL-4 (Figures 5(a) and 5(d)) and GR (Figures 5(b) and 5(e)) were localized in different compartments of the identical cells (Figures 5(c) and 5(f)), which are neurons, of the LC.

#### 3.2.5. Expression of TH

**Figure 6:**
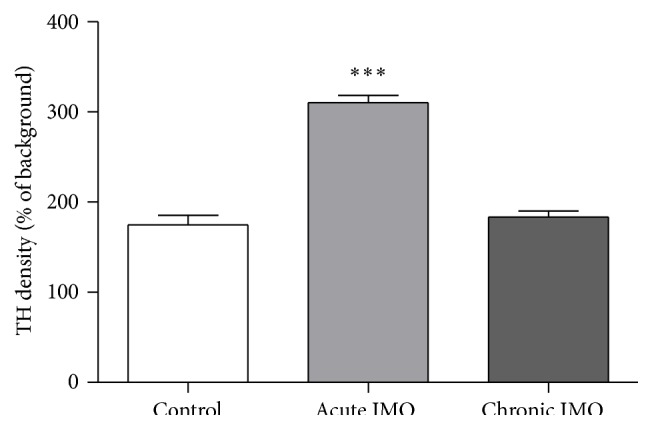
Mean (± SEM) quantification of TH expression in the LC of the control group and groups subjected acute and chronic IMO. Acutely stressed rat expressed significantly increase in TH density whereas chronically stressed animals showed no change. (*n* = 4-5 per group) ^*∗∗∗*^ *P* < 0.001 versus control; ANOVA.

Immunofluorescence was performed to quantify the expression of the TH in the LC of the controls and the stressed rats (*F*_2,10_ = 43.2, *P* < 0.001). Density of TH was considerably increased in the acute IMO group compared to control group (*P* < 0.001; Figure 6), but no significant alteration was observed in the chronic IMO group.

## 4. Discussion

It has been reported that IMO-induced stress had an inhibitory effect on IL-4 release in peripheral tissues of human and rat [28–30]. Lipopolysaccharide (LPS) had a similar effect of stress in respect to inducing immunomodulation characterized by increased production of proinflammatory cytokines. IL-4 protein level was considerably decreased in the LPS-treated rat brain and IL-4^−/−^ mice were more vulnerable to LPS than wild type [4, 31, 32]. In this study, it was demonstrated that the production of IL-4 protein in the LC region of the brain was significantly decreased in the acutely stressed group. Although it has been reported that there was no significant change or elevated release of peripheral IL-4 in stress responses [8, 33, 34], these controversial data indicated that stress-induced alteration of IL-4 secretion may differ in a tissue-specific manner and further studies should be demonstrated. Taken together, these findings suggest that stress has an inhibitory effect on IL-4 production in the brain.

It has been reported that IL-4 is released from microglia in the cortex of the brain [12]. Also an* in vitro* study reported glia as the cell source of IL-4 in the brain [4]. In this study, however, IL-4 was produced from the TH-producing cells identified as noradrenergic neurons [26] of the LC. Neither astrocyte nor microglia released IL-4 in the LC region of the brain. The data is the first to identify IL-4 expressing cells in the LC suggesting that IL-4 is produced by different type of cells in a site-specific manner in the brain. Further research is required to demonstrate identification of IL-4-secreting cells in other brain regions besides the cortex and the LC.

Previous work has shown that restraint stress-submitted groups were more anxious than nontreated groups [35–37] and increment of corticosterone had an anxiolytic-like effect in animal model [38]. Central and peripheral IL-4 concentration had an effect of suppressing LPS-induced sickness behavior [39, 40] and IL-4^−/−^ mice exhibited more anxious behavior compared to wild type [41]. It has been reported that CORT had a suppressive effect on IL-4 protein production and signaling in* in vitro* and* in vivo* studies [22, 23, 42, 43]. It was observed that IMO stressed rats had significantly increased anxiety and reduced IL-4 protein level in the brain. Reduction of IL-4 protein release from LC was accompanied by an increase of circulating CORT level in acute stress response. GR is known to be abundantly expressed in the LC neurons [44] and it was confirmed that GR and IL-4 were localized in the identical neurons of LC in this study. Consequently the data suggests that acute IMO-induced hypercorticism may contribute to downregulation of IL-4 production in the LC and this decrement of IL-4 level leads to behavioral change.

Accumulating evidence suggests that a single exposure of stress leads to a significant increase in circulating CORT level and nuclear GR intensity and activity [45] as a result of HPA-axis activation [13, 14, 46]. It has been reported, however, that chronic exposure of homotypic stress induces hypocorticism [45, 47–50]. Also increment of nuclear GR intensity and activity was abolished in the chronic restraint stressed group [45]. In the present study, CORT concentration was increased in the acute IMO group, whereas chronic stressed rats had decreased CORT level compared with controls. Taken together, the findings suggest that chronically prolonged stress impaired glucocorticoid negative feedback leading to HPA-axis hypoactivity. Since CORT has an ability to inhibit IL-4 production [22, 23, 42, 43] and to increase TH expression [38], chronic stress-induced hypocorticism might reverse the effects produced by acute stress.

Single exposure of stress or lipopolysaccharide has been shown to stimulate release of NE or to increase TH mRNA level and TH activity [17, 51–53]. In contrast with acute stress, TH mRNA level was decreased in a chronic mild stressed group [54]. Since TH is a rate-determining enzyme of NE synthesis, change of TH protein was not detected right after exposure of acute stress [55]. Stress also has shown to increase TH immunoreactivity, which is consequence of enhanced enzyme activity and TH protein concentration [56]. In the present study, acute IMO induced increase of TH-immunoreactivity in the LC, suggesting this increment might be result from increase of enzyme activity.

In conclusion, stress-induced decline of IL-4 concentration from LC neurons may be related to anxiety-like behavior and an inverse relationship exists between IL-4 secretion and HPA/SAM-axes activation. These data suggest that modulation of these signaling factors, cytokine, catecholamine, and CORT is required to adapt to homeostatic mechanisms in response to stressful events.

## Abbreviations

CNS: Central nervous system
CORT: Corticosterone
EPM: Elevated plus maze
ELISA: Enzyme linked immunosorbent assay
FITC: Fluorescein isothiocyanate
HPA-axis: Hypothalamic-pituitary-adrenal-axis
IMO: Immobilization
IL-4: Interleukin-4
LPS: Lipopolysaccharide
LC: Locus coeruleus
NE: Norepinephrine
SAM-axis: Sympathetic-adrenal-medullary-axis
TH: Tyrosine hydroxylase.
